# Understanding how management can prevent degradation of the structurally fragile soils of the Amazonian periphery

**DOI:** 10.1016/j.eja.2023.127037

**Published:** 2024-02

**Authors:** Jéssica de Freitas Nunes, Lorena Silva Campos, Alana das Chagas Ferreira Aguiar, Sacha Jon Mooney, Karina Andrade Pimentel, Emanoel Gomes de Moura

**Affiliations:** a Postgraduate Program in Agroecology, Maranhão State University, São Luis, Maranhão 65000-000, Brazil; b Department of Biology, Federal University of Maranhão, São Luís, Maranhão 65080- 805, Brazil; c School of Biosciences, University of Nottingham, Sutton Bonington Campus, Loughborough LE125RD, United Kingdom

**Keywords:** Soil organic carbon stabilization, Base cations retention, Tropical agrosystem, Sustainability

## Abstract

The dynamics and responses to mulching management processes, which affect sustainability in tropical agroecosystems, remain poorly understood. Therefore, this study aims to evaluate and distinguish the short-and long-term effects of mulch of leguminous biomass on fertility of a tropical soil enriched with calcium. This experiment was conducted using the treatments: Long-term mulching (LTM) consisted of planting without mulch in 2019 in soil that had been mulched for six years previously, while short-term mulching (STM) consisted of planting without mulch for six years and with mulch only in 2019. LTM + STM consisted of planting in mulched soil for seven years (from 2013 to 2019), while LTM + synthetic nitrogen (LTM + sN) consisted of the LTM treatment with the addition of 150 kg N ha^−1^. The remaining treatments were STM + sN; LTM + STM + sN; bare soil with sN, and bare soil without sN as control. In areas with LTM the interactions between products derived from biomass, sN, and Ca resulted in higher total SOC and BS, while STM maintained soil moisture, decreased penetration resistance, and enhanced N uptake providing biological nitrogen able to replace sN for maize nutrition. The positive effects of short- and long-term mulching were cumulative as they increased accumulated N by maize in 163%, and maize grain yield by 125% (4.77–10.78 Mg ha^−1^) compared to cultivation with sN without mulch. Our results showed that interactions between continuos mulch of leguminous biomass, Ca and sN prevent degradation of agricultural land in Amazonian conditions. Therefore, this combination must be recommended to prevent Amazonian soil management, which in turn reduces the risk of new deforestation in Amazonian periphery.

## Highlights

• Mulch prevented land degradation, but short- and long-term effects were different.
• Continuous mulching contributed 54% more than urea alone to N harnessing.
• Long-and short-term mulching effects were cumulative, increasing grain yield by 125%.

## Graphical Abstract

**Figure:**
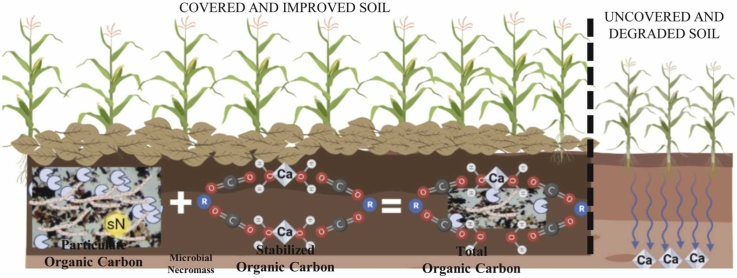

## 1 Introduction

In many regions of the humid tropics, such as the Amazonian periphery, the sustainability and feasibility of agroecosystems are predominantly dependent on a balance between soil organic matter (SOM) input/output and the maintenance of an adequate base saturation (BS), on the rooting zone (Quesada et al., 2020). In this region, land degradations and deforestation of other areas occur when soil management cannot overcome the natural forces that decrease the SOM content due to fast turnover and reduce the BS due to high leaching losses of nutrient cations (Ramos et al., 2018). Maintaining or improving soil organic matter (SOM) in tropical regions is a major challenge due to high rates of biomass decomposition. Agricultural systems that combine low soil tillage and high inputs of plant residues, such as pruning from trees or shrubs with high biomass production, have shown promise as effective alternatives for ensuring tropical soil productivity (Barreto et al., 2012, Partey, 2011).

Furthermore, most soils in this region have a low intrinsic resilience against physical degradation due to the low content of aggregator elements such as elemental iron, soil carbon and calcium (Moura et al., 2018). In the natural conditions of the Amazonian periphery, the soil-vegetation system is resilient against degradation due to complete soil surface cover combined with the high activity of the soil fauna (Ciemer et al., 2019). Therefore, in tropical agroecosystems, strategies using soil cover with biomass application has been specially recommended as an alternative for the management of tropical soil (Iqbal et al., 2020).

In the short-term, mulch is thought to maintain soil moisture and delay hardening even under high evaporation rates. However, though positive short-term mulching effects on crop yields has been verified, it has been often variable under irregular rainfall conditions (Moura et al., 2017). Furthermore, the short-term effects of mulch have not been able to contribute to replacing shift cultivation in the humid tropics due to its reduced effect on the key drivers of sustainability of tropical soil use (López-Carr, 2021).

Long-term mulching with leguminous tree residues has been suggested as a method for maintaining a balance between C inputs and outputs over time, increasing soil organic carbon (SOC) (Iqbal et al., 2020) with a constant supply of high-quality biomass from the fast-growing leguminous trees (Coelho et al., 2018). However, the long-term leguminous residue mulching may not always be able to increase SOC significantly in tropical conditions if carbon use efficiency is low and most of the carbon applied via biomass is not converted into stabilized SOC (Sena et al., 2020). Indeed, despite the possible positive effects of leguminous biomass mulch on soil improvements and crops not all evidence points in the same direction; mainly because the mulching effect does not always manage to increase SOC stabilization and to decrease basic cations leaching, which are key points to the sustainability of tropical agrosystems (Yang et al., 2021).

The long-term stability of SOC dependent on the wide array of processes that control SOM turnover. Recent works (Liang et al., 2017, Högberg et al., 2020) have highlighted that the mechanisms through which SOC can be biologically stabilized depend on the capacity of the biomass added to increase the microbial necromass to interact with available polyvalent cations in soil (Liang et al., 2020). In addition, contribution of microbial necromass to stabilized SOC can make up more than 60% of SOC in crop lands (Liang et al., 2019).

To increase carbon use efficiency microbial substrate with high quality has been suggested to decrease anabolism/catabolism ratio, when then, more microbial residues, and less CO_2_ is produced per amount of plant litter metabolized and stable SOM stocks are predicted to be relatively high (Castellano et al., 2015). For the same reason, anthropogenic N input has also been reported as another mechanism for SOC stabilization or accumulation by increasing microbial necromass in croplands (Hu et al., 2022). In a way that, to take full advantage of the use of mulching it is important to identify how complementary management practices can potentialize the drivers’ mechanisms by which mulching, when used in short term or in long-term, ensure sustainability, replace shifting cultivation and avoid deforestation in humid tropic (Kader et al., 2017).

We hypothesize that the knowledge of the differences between short-and long-term mulching can contribute to improving the management of leguminous tree biomass in the humid tropics to prevent soil degradation by maintaining SOC and BS at adequate level, and increasing stabilized mineral associated organic carbon (MAOC) fraction. Therefore, this study aims to evaluate and distinguish the short-and long-term effects of mulch on SOC accumulation when high-quality leguminous biomass is applied alongside synthetic N (sN) to increase interactions between products derived from biomass and polyvalent cations in soil enriched with calcium. We also assess how these processes contribute to BS maintenance and physical improvement of a hard-setting tropical soil, as well as its influence on grain yield in maize.

## 2 Materials and methods

### 2.1 Experimental area and setup

The experimental work was conducted in the municipality of São Luís, Maranhão, Brazil, (2°30′S, 44°18′W)). The region’s Köppen climate classification is tropical savanna climate with dry-winter characteristics (Aw) type. The soil of the study area was classified as an Arenic Hapludult with hard-setting characteristics due to the typical relationship between penetration resistance and volumetric water content (Moura et al., 2009).

### 2.2 History and layout of the experimental area

The experimental area had been fallow and overgrown with native herbs and shrubs since 2008 and was cleared in August 2014. Following mowing in September 2014, lime was surface applied to the site at a rate of 1 Mg ha^−1^, corresponding to 390 kg ha^−1^ Ca and 130 kg ha^−1^ Mg. In the same period, natural gypsum (an agglomerating mineral composed of hydrated calcium sulfate (CaSO_4_•2 H_2_O) produced in local deposits from Cretaceous marine formations) was applied at a rate of 6 Mg ha^−1,^ which corresponds to 1010 kg/ha Ca. The grain size of the gypsum was such that 95% by weight passed through a 0.25 mm screen mesh. Maize (cultivar AG 1051) was sown in October of each year between 2013 and 2018, with a between-row spacing of 80 cm and 25 cm between plants. The fertilizer application prior the sowing consisted 55 kg P ha^−1^ as triple superphosphate, 100 kg K ha^−1^ as potassium chloride, and 5 kg Zn ha^−1^ in the form of zinc sulfate.

### 2.3 Experimental design this experiment

This experiment was conducted under a no-tillage system using a randomised block design with the following eight treatments:

Long-term mulching (LTM): planting without mulch in 2019 in soil that was mulched with dry biomass of *Gliricidia sepium* at a rate of 12 Mg ha^−1^ year ^−1^ for six years (2013 – 2018).

Short-term mulching (STM): planting with 12 Mg ha^−1^ of dry *Gliricidia sepium* biomass in 2019 on soil that had not been mulched for six years (2013 – 2018).

LTM plus STM (LTM+STM): planting with 12 Mg ha^−1^ of dry *Gliricidia sepium* biomass in 2019 in soil which that had been mulched with *Gliricidia sepium* at a rate of 12 Mg ha^−1^ year ^−1^ for the six preceding years (2013 – 2018).

LTM plus synthetic nitrogen (LTM+sN): LTM plots with sN applied at a rate of 150 kg N ha^−1^, in three applications - 50 kg at the time of planting, and 50 kg at 25 and 44 days after emergence of the maize. This pattern of N application was used on all sN plots.

STM plus synthetic nitrogen (STM+sN).

STM LTM plus STM plus synthetic nitrogen (LTM+STM+sN).

Bare soil with nitrogen (sN).

Control (C): bare soil without nitrogen.

Biomass was taken from *G. sepium* trees growing in rows used as windbreaks around the experimental plot and applied at a normal rate for modified alley cropping systems per Aguiar et al. (2010).

Total of applied organic Ca in plots with LTM + STM, was 1080 kg ha^−1^ and total of applied Ca in these plots was 2490 kg ha^−1^. Maize (cultivar 30F35) was planted on October 2, 2019, with a between-row spacing of 80 cm and 25 cm between plants. At the time of planting, fertilizer at a rate of 55 kg P ha^−1^ as triple superphosphate, 83 kg K ha^−1^ as potassium chloride, and 5 kg Zn ha^−1^ in the form of zinc sulfate was manually applied. In treatments with sN, 50 kg sN ha^−1^ was applied as urea. In addition, the latter plots were fertilized at 25 and 44 days after emergence of the maize plants using 50 kg ha^−1^ of sN as urea in each fertilization. The plants were irrigated at an interval of seven days, for a total application of 350 mm over the entire growing period. Water was supplied by drip tape irrigation using one tape per row with emitters spaced 25 cm apart. Throughout 5 h each drip emitter delivered 1.25 L h^−1^, which was equivalent to 25 mm of water per irrigation.

### 2.4 Organic matter and soil chemical analyses

Soil samples for chemical and SOM analyses were collected in 2019 at depths of 0–20 cm. Using a Dutch auger with a 3-inch diameter three replicates per plots were collected. SOM was physically fractionated following Cambardella, Elliot (1992). Soil MAOC was calculated as the difference between total organic carbon (TOC) and particulate organic carbon (POC). The TOC stock (TOCS) of the 0–30 cm layer was calculated using Eq. 1:

(1) $$\mathrm{TOCS}=\frac{\mathrm{TOC}\;x\rho s\;x\;E}{10}$$

TOCS = organic C stock at a given depth (Mg ha^−1^); TOC = total organic C content at the sampled depth (g kg^−1^); ρs = soil bulk density (kg dm^−3^); E = thickness of the layer (20 cm). In order to construct the graph of the soil structure quality was used the criterion defined by Johannes et al. (2017) using MAOC due to its key role in the sustainability soil use (Lavallee et al., 2019).

We furthermore determined according to Raij et al. (1986), the sum of bases cations (SBC = K^+^ + Ca^2+^ + Mg^2+^) and cation exchange capacity (CEC = K^+^ + Ca^2+^ + Mg^2+^ + H^+^ + Al^3+^) which were used to calculate base saturation BS = [(SBC⁄ CEC) x 100]. For determination was used a Varian 720-ES ICP Optical Emission Matter Analysis Spectrometer. The accumulations of Ca, in kg ha^−1^, in the 0–20 cm layer of the soil profile, were calculated according to the Eq. 2:

(2) SCaA=SEC xρs x E x10

where, SCaA = Soil Ca accumulated in the 0–20 cm layer (kg ha^−1^); SEC = soil element content (mg kg^−1^); ρs = soil bulk density (Mg m^−3^); *E* = thickness of the layer (m). In order to construct the graph of the base saturation the table with a criterion of leaching defined by Hazelton and Murphy (2007) was used.

### 2.5 Soil physical properties

Soil moisture content and soil penetration resistance, as an indicator of soil rootability, were measured at depths of 0–0.20 m with three replicates per plot, in 2019, after seven days without irrigation. The soil penetration resistance was measured using a digital penetrometer (Falker, Porto Alegre, Brazil) with 5 cm graduations. In order to plot soil penetration resistance, the table of critical levels defined by Hazelton and Murphy (2007) was used. Soil moisture content was measured in 2019 using the gravimetric method with undisturbed soil samples obtained at three points along a given line in volumetric rings with a 100 cm^3^ volume. The same samples were used to determine soil bulk density (ρs). Three replicates were collected per repetition at each plot. The soil ρs was calculated as m/v, where m is the dry collected soil mass at 105 °C, and v is the ring volume (Thomasson, 1978).

### 2.6 Plant analysis

Plant dry matter and total nitrogen content were measured at two physiological periods of maize: at tasseling and at the physiological maturity stage, according to the standard distillation method described by Cottenie (1980). The various parameters related to nitrogen in the maize plant were calculated according to (3), (4), (5), (6), (7):

(3) $$\mathrm{NT}=\mathrm{DM}\;x\;N_{\mathrm{tasseling}}\;x1000$$

(4) $$\mathrm{ANTP}=N_{\mathrm{total}}-(N_{\mathrm{tasseling}}\;x1000)$$

(5) $$\mathrm{TAN}={\mathrm{DM}}_{\mathrm{maturity}}\;x\;N_{\mathrm{maturity}}\;x1000$$

(6) $$\mathrm{CNU}=\frac{N_{\mathrm{uptaken}}-N_{\mathrm{uptaken}\_\mathrm{sN}}}{N_{\mathrm{uptaken}}}$$

(7) $${\mathrm{CNU}}^{*}=\frac{N_{\mathrm{uptaken}}-N_{\mathrm{uptaken}\_\mathrm{control}}}{N_{\mathrm{uptaken}}}$$

NT = Accumulated nitrogen at tasseling (kg ha^−1^);.

DM = dry matter (kg ha^−1^);.

N_tasseling_ = N at tasseling (g kg^−1^);.

ANPT = Accumulated nitrogen post-tasseling (kg ha^−1^);.

N_total_ = total nitrogen (kg ha^−1^);.

TAN = Total accumulated nitrogen (kg ha^−1^);.

DM_maturity_ = total dry matter at maturity (kg ha^−1^);.

N_maturity_ = N at maturity (g kg^−1^);.

CNU = Contribution to nitrogen uptake - for treatments with applied sN (%);.

N_uptaken_ = uptaken N in the treatment (kg ha^−1^);.

N_uptaken_sN_ = uptaken N in the treatment with sN alone (kg ha^−1^);.

CNU* = Contribution to nitrogen uptake - for treatments without applied sN (%);.

N_uptaken_control_ = uptaken N in the control treatment (kg ha^−1^).

The harvest was carried out manually in early June 2019, when the plants were at physiological maturity. Kernel weight (100 kernel), and maize grain yield (MGY) were assessed in a 10 m² area. MGY was determined and standardized using a moisture level of 145 g kg^−1^.

### 2.7 Statistical analyses

The data were analyzed using the ESTATISTIX program (version 9). An analysis of variance (ANOVA) was undertaken on all data, after which the means were compared using Fisher’s LSD test at a level of 5% probability of error**.** The software SIGMAPLOT (version 11.0) was used to plot the graphs. Correlations between the SOC fractions, basic cations, maize grain yield, and soil penetration resistance were investigated through canonical redundancy analysis (RDA).

## 3 Results

### 3.1 Changing in soil organic carbon and sum of base cations by mulching and synthetic N

**Fig. 1:**
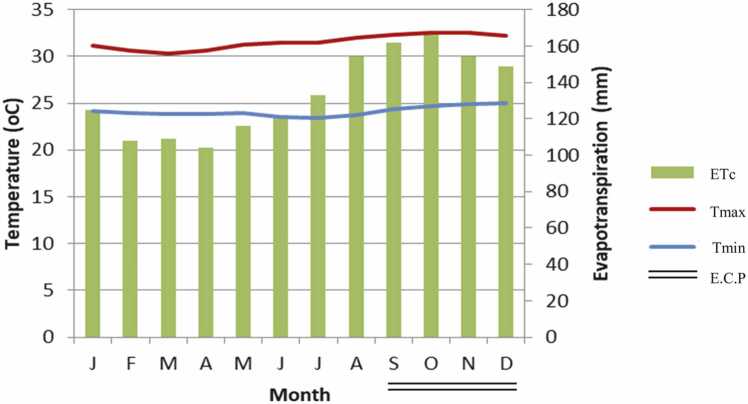
Reference temperature and evapotranspiration graph from January to December 2019. ETc means corn evapotranspiration; **E.C.P** means period of conducting the experiment.

**Fig. 2:**
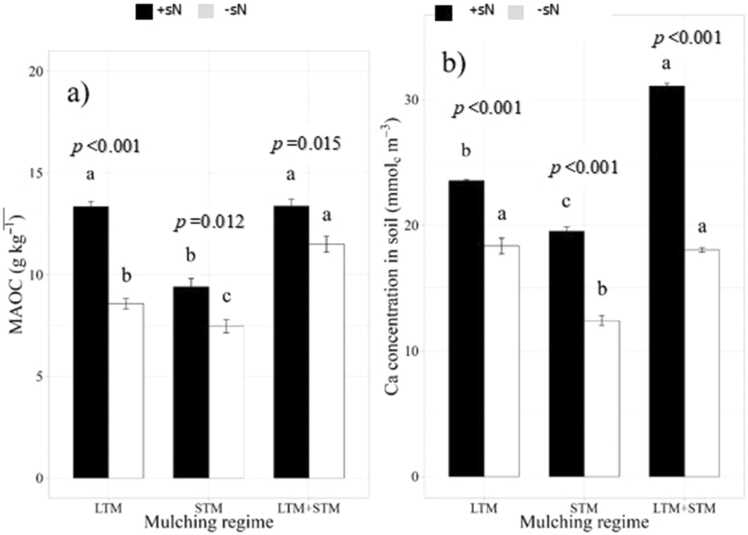
Effects of mulching regime x synthetic nitrogen (sN) application interaction on (a) MAOC (n = 4) and (b) Ca concentration in soil (n = 4). Bars sharing the same letter within each sN application do not differ significantly (Tukey’s test, *p* ≤ 0.05). F-test was used to compare sN application within each mulching regime. Error bars represent standard deviation.

Both STM and LTM treatments had higher TOC than the control, though the increase in STM was 15% lower than in LTM (Table 1). There was a positive effect of sN on TOC, but only when it was used in areas that had received LTM. Indeed, in LTM + sN treatment TOC was 50% higher than in plots with only LTM, and 18% higher than in STM+sN. Larger contribution to differences in TOC was given by MAOC, which was higher in LTM + N than in LTM + STM but there was no significant difference between STM+N and LTM+STM treatments. Mulching and sN doubled TOCS in LTM+STM+sN in the 0–20 cm layer compared to the control (57.70 Mg ha^−1^ vs. 28.20 Mg ha^−1^, respectively) (Fig. 1a, b). According to criteria established by Johannes et al. (2017) only the treatments which received long-term mulching and nitrogen were able to maintain soil structure in very good range (MAOC:clay ratio > 1/8), while STM+ N and LTM were in the good range and treatments STM and without biomass were in degraded range (Fig. 2a).

**Table 1:** Soil properties at the beginning of the experiment.

pH, CaCl_2_	4.0
Soil organic matter, mg kg^−1^	20
Phosphorus, mg dm^−3^	15
Potencial acidity (H + Al), mmol_c_ dm^−3^	25
Calcium, mmol_c_ dm^−3^	15
Magnesium, mmol_c_ dm^−3^	9
Potassium, mmol_c_ dm^−3^	1
Cation exchance capacity (CEC), mmol_c_ dm^−3^	50
Percentage base saturation, %	50
Coarse sand, g kg^−1^	300
Fine sand, g kg^−1^	545
Silt, g kg^−1^	61
Clay, g kg^−1^	90

In general, SBC followed the same tendency of the TOC, with SBC 33% lower in STM compared to LTM (Table 2). Again, the effect of sN was only significant in the LTM plots. SBC was higher in LTM+sN than in all other treatments except for LTM+STM+sN, where it was 33% higher than the former treatment. There was no difference in SBC between STM+sN and LTM+STM. Meanwhile, soil Ca accumulated (SCaA) in LTM+STM+sN after six years was approximately half of the applied Ca (2490 kg ha^−1^ to 1240 kg ha^−1^). In contrast, the control area lost 80% of the applied Ca (1410 kg ha^−1^ to 292 kg ha^−1^). Therefore, this treatment can be consider strongly leached according to Hazelton and Murphy (2007) criteria (Fig. 2b). According to the same criteria STM + sN treatments were moderately leached, LTM + STM + N very weakly leached and other treatments all weakly leached.

**Table 2:** Results of soil organic carbon fractions analyses, soil organic carbon accumulated and mineral associated organic carbon:clay ratio after seven years of soil management.

SOC fractions	Treatments							
	LTM+STM+sN	LTM+STM	LTM+sN	STM+sN	LTM	STM	sN	Control
POC (g Kg^−1^)	10.3 a	7.5 b	6.6c	7.2 b	6.2 bc	5.9c	3.3 d	3,1 d
MAOC (g Kg^−1^)	13.8 a	11.4 b	13.1 a	9.5c	8.9 d	7.1 d	6.5 e	5,9 f
TOC (g Kg^−1^)	24.0 a	18.9c	19.7 b	16.7 d	15.1 e	13.1 f	9.8 g	9,4 g
TOCS (Mg ha^−1^)	57.7 a	49.1c	51.2 b	43.4 d	40.6 e	34.0 f	29.6 g	28,2 g
MAOC:Clay	1/7	1/9	1/7	1/10	1/11	1/14	1/15	1/17

Values followed by different letters in the line indicate a significant difference at the 5% level by Tukey test. LTM = long-term mulch: STM = short-term mulch; sN = synthetic nitrogen; POC = particulate organic carbon; MAOC = mineral associated organic carbon; TOC = total organic carbon: TOCS = total organic carbon stock: MAOC:Clay = mineral associated organic carbon fraction: clay ratio.

### 3.2 Soil humidity and soil penetration resistance

**Fig. 3:**
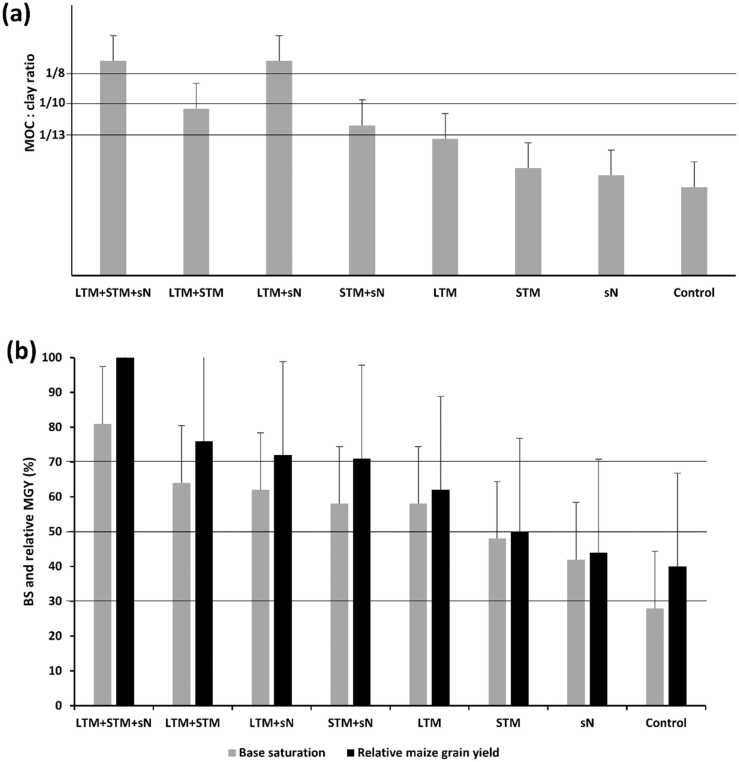
Mineral associated organic carbon:clay ratio after seven days of soil management (a). Relative maize grain yield from the 2019 harvest and base saturation after seven days of soil management (b). LTM = long term mulch; STM = short term mulch; sN = synthetic nitrogen. Error bars represent standard desviation.

The results of soil water content showed that different mulching regime and nitrogen treatments affected water maintenance in the root zone. After seven days without irrigation, soil water content was higher than the critical level of 5% base mass in the 0–12.5 cm layer in the LTM+STM and LTM+STM+sN treatments and was lower than 3% in the N and control treatments (Fig. 3a). The effect of sN additions on water content is apparent in the soil moisture results, with LTM+sN and STM+sN always having higher moisture values than LTM and STM, respectively. Both mulching treatments decreased soil penetration resistance (SPR), but this positive effect was more accentuated in STM than in LTM (Fig. 3b). After seven days without irrigation, the soil was very dense in the 6–20 cm layer in the N and control plots, while SPR did not achieve the threshold of 2 MPa in the 0–20 cm layer in all treatments with STM. In LTM and LTM+sN, only the 11–20 cm layer was very dense and higher than 2 MPa.

### 3.3 Amount of nitrogen accumulated maize yield and principal components analysis

TAN in STM was 18% higher than in LTM, though additions of sN in the post-tasseling stage these treatments increased this difference to 47% (Table 3). TAN in LTM+STM was higher than in LTM+sN and was not significantly different from STM+sN. The TAN in LTM+STM+sN was 38% higher than STM+sN. When used alone, values of the contribution for N uptake (CNU) for urea were very low (8%), as opposed to 62% for LTM+STM+sN, which was the highest of all treatments. The contribution of LTM+sN also was low (25%) compared to 45% for STM+sN. There was no significant difference between the treatments LTM+STM, LTM+sN, and STM for this variable. Table 4.

**Table 3:** Soil chemical analyses and soil accumulated calcium content; sum of base cation and base saturation after seven years of soil management.

Cations content	Treatments							
	LTM+STM+sN	LTM+STM	LTM+sN	STM+sN	LTM	STM	sN	Control
Ca (mmol_c_ m^−3^)	31.4 a	18.3 d	23.5 b	19.4c	18.8 d	12.2 e	10.2 f	7.4 g
SCaA (Kg ha^−1^)	1.240 a	723 e	928 b	766c	742 d	482 f	403 g	292 h
Mg (mmol_c_ m^−3^)	14.6 a	7.0c	10.5 b	5.7 d	4.0 e	2.8 f	2.3 f	0.7 g
K (mmol_c_ m^−3^)	5.1 a	3.3c	4.3 b	2.3 d	3.9c	0.4 e	0.4 e	0.5 e
SBC (mmol_c_ m^−3^)	51.0 a	28.6c	38.3 b	27.4 d	26.7 e	17.9 f	12.9 g	8.5 h
BS (%)	81 a	64 b	62 b	58 b	58 b	48c	43 g	28 h

Values followed by different letters in the line indicate a significant difference at the 5% level by Tukey test. LTM = long-term mulch; STM = short-term mulch; sN = synthetic nitrogen; SCaA = Soil accumulated calcium content; SBC = Sum of base cation; BS = Base saturation.

**Table 4:** Analyses of nitrogen content in maize plants, kernel weight and maize grain yield from the 2019 harvest.

Variables	Treatments							
	LTM+STM+sN	LTM+STM	LTM+sN	STM+sN	LTM	STM	sN	Control
ANPT (Kg ha^−1^)	51.75 a	47.21 b	38.21 d	41.48c	37.74 e	33.17 f	13.26 g	22.21 h
TAN (Kg ha^−1^)	159.l3 a	112.1c	104.4 d	115.6 b	94.9 e	80.6 f	70.6 g	55.6 g
CNU (%)	62 a	50 b	42c	48 b	45c	25 d	8 e	--
KW (g/100)	30.7 a	28.4 b	28.3 b	28.2 b	25.2c	21.6 d	18.5 e	15.5 f
MGY (Mg ha^−1^)	10.8 a	8.2 b	7.8c	7.7c	6.7 d	5.6 e	4.8 f	4.4 g

Values followed by different letters in the line indicate a significant difference at the 5% level by Tukey test. LTM = Long term mulch; STM = Short term mulch; sN = synthetic nitrogen; ANPT = Accumulated N post-tasselling; TAN = Total accumulated nitrogen; CNU = Contribution for nitrogen uptake; KW = Kernel weight; MGY = Maize grain yield.

**Fig. 4:**
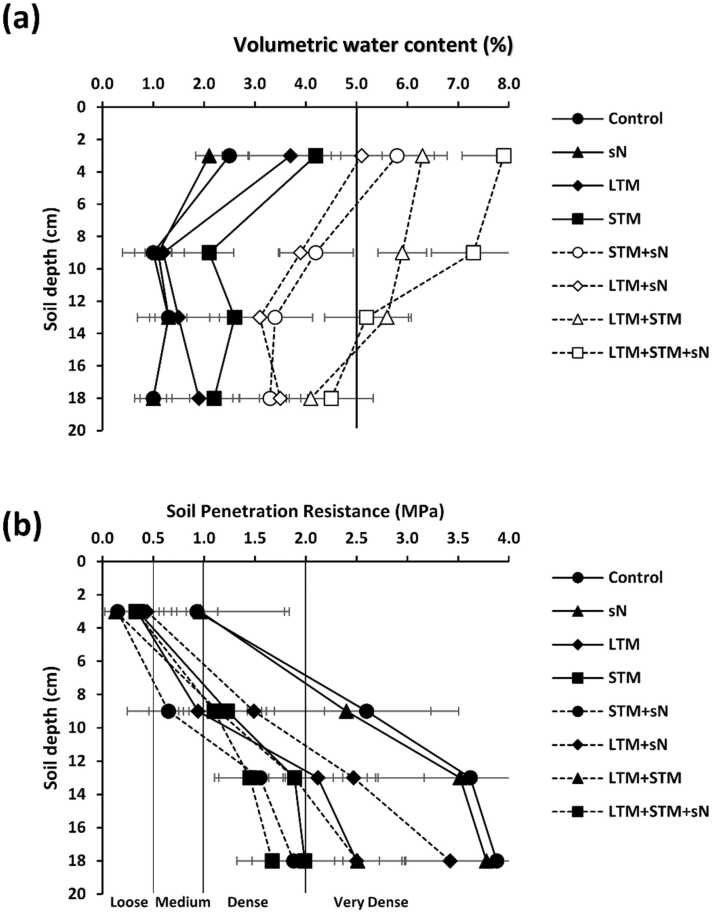
Soil humidity (a) and Soil Penetration Resistance (b) seven days after irrigation. Vertical bars show the critical levels of Moura et al. (2009); Hazelton and Murphy (2007). LTM = long term mulch; STM = short term mulch; sN = synthetic nitrogen. Error bars represent standard deviation.

**Fig. 5:**
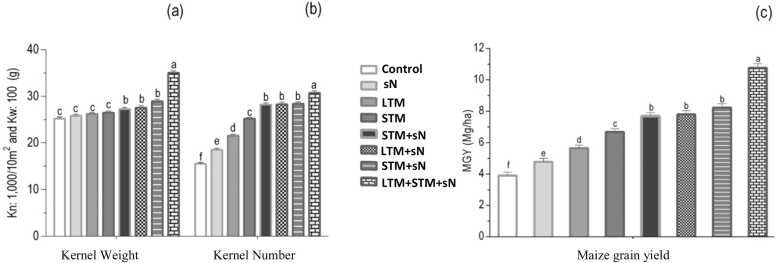
(a) Kernel number (a), kernel weight (b), and grain yield (c) of maize in the experimental area in 2017. LTM+STM+N = soil with long-term mulching, covered by mulch and with added nitrogen; LTM+STM = soil with long-term mulching and covered by mulch; LTM+N = soil with long-term mulching and with nitrogen; STM+N = soil covered by mulch and with nitrogen; LTM = soil with long-term mulching; STM = soil covered by mulch; N = soil with nitrogen and control. Different letters indicate differences at the 5% level by Tukey test. Error bars represent standard deviation.

The kernel weight was significant in treatments with mulches or sN alone, in the following order: STM > LTM > sN > control (Fig. 4). The difference was most pronounced between LTM+STM+sN and sN, with the former 109% higher. The effects of long-and short-term mulching on MGY were positive, and the size of the effect was dependent on sN. When sN was used, there was no difference in MGY between the two mulching types (LTM+sN = STM+sN), though MGY was higher in STM than in LTM without sN. The application of urea alone increased MGY by 22%. There was no significant difference between LTM+STM and LTM+sN. In LTM+STM+sN, MGY was 20% higher than in LTM+sN. The principal components analysis showed a cluster with Ca, POC, MAOC, and MGYin the treatments with mulches and Ns, mainly in LTM + sN. On the other hand, POC was more associated with STM + sN (Fig. 5).

## 4 Discussion

### 4.1 Mulches, Ca and synthetic N interactions influence in soil organic carbon and sum of base cations

**Fig. 6:**
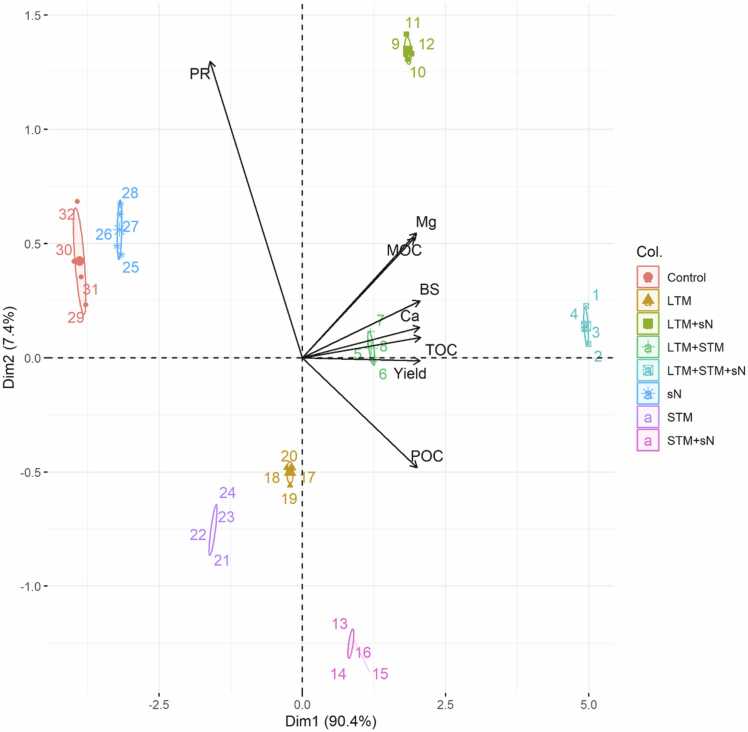
Soil physical-chemical properties, and yield responses to treatments. MAOC: Mineral Organic Carbon. POC: Particulate organic carbon. TOC: Total Organic Carbon. Ca: Calcium. Mg: Magnesium. BS: Bases Sum. PR: Penetration Resistance.

SOM dynamics involving mulch of biomass and sN, as well as their multiple interactions in a tropical environment, are complex (Poffenbarger et al., 2017). However, we have shown clear evidence that the interactions between mulches of leguminous biomass with sN were responsible for the largest increase in SOC. First, TOC was not different in the sN and control treatments, but in treatments combining sN with STM or LTM, TOC was 82% and 109% higher than control, respectively. Second, the increase of TOC in mulching treatments without sN was lower (39% and 60%). Fig. 6.

The difference of only 22% in TOC between LTM+STM+N and LTM+N can be accounted for by the saturation concept proposed by Stewart et al. (2007), which notes that there is a maximum equilibrium C level that will be attained when C input is maximised.

In addition, a larger contribution to increased SOC was given by MAOC fraction, which is protected from decomposition through association with soil minerals (Ellerbrock and Gerke, 2018). Mineral associations include chemical bonds between SOC and mineral, which makes MAOC less accessible to decomposers and with greater degree of stabilization (Lavallee et al., 2019). In a sandy loam soil like the one present in this experiment without the physical protection mechanisms provided by clay, it is more likely that the stable compounds comprising MAOC have been largely transformed through biological activities.

This biological activity is speed up by sN, increasing microbial necromass (Buckeridge et al., 2020). In soils with an exchange complex dominated by Ca^2+^ (Von Lützow et al., 2006) stated mechanisms that promote microbial transformations that lead to the creation of recalcitrant organic products through The formation of polyvalent cation bridges due to interaction with microbial necromass. Indeed, an increase of 93% in Ca content between LTM+sN and LTM (23.5 mmol_c_ m^-3^ vs 12.2 mmol_c_ m^-3^, respectively) could be explained by the retention of Ca during SOC transformation in the presence of sN (Rowley et al., 2018). Moreover, as a result of this process, the POC:MAOC ratio decreased from 1.0:1.2 in LTM to 1.0:2.0 in LTM+sN, which reflects a proportional increase in MAOC due to sN influence. In the same way, when sN was added in LTM+STM, Ca was increased by 72% (18.3 mmol_c_ cm^-3^ to 31.4 mmol_c_ cm^-3^). These results suggest that increased TOC and Ca maintenance in the root zone resulted from the interaction between increased necromass, Ca, and sN, which occurs in the soil environment created by long-term mulching and improved maize productivity (Sena et al., 2020). The large difference in TOCS between LTM+STM+sN compared to the control (57.70 Mg ha^-1^ vs 28.20 Mg ha^-1^) shows the high SOC stabilisation capacity of soil due to a no-till system and increased Ca and N availability (Stewart et al., 2017). Furthermore, the principal components analysis showed Ca and organic carbon accumulation are significantly associated in treatments with mulch and sN.

The criteria used for soil evaluation (Johannes et al., 2017, Hazelton and Murphy, 2007) were able to define whether its destination would be improvement or degradation, depending on management practices. Thus, the contribution of mulching to avoid soil degradation over time can be seen comparing LTM (with good structure and weakly leached) to the control treatment (degraded and strongly leached) after six years of gypsum and lime application. On the other side, if just one year of application of biomass mulch of legume is insufficient to avoid soil degradation when STM was degraded and moderately leached, only one year without mulch in LTM took the soil close to the limit for degradation (MAOC: clay ratio = 1/11), and to 10% lower base saturation compared to LTM + STM (58–64%). Therefore, continuously biomass application is mandatory if sustainability is the objective to be achieved in the context of the agrosystems of humid tropic.

### 4.2 Physical improvements, N uptaken and maize grain yield

Regular irrigation and the no-tillage system used in this experiment should be considered when analysing the results of the improvement in the physical conditions of the soil due to their positive influence as interactive factors for the accuracy of data.

The effect of the interactions between continuous mulch application, Ca and nitrogen not only prevented degradation in LTM + STM + sN but also improved soil conditions for roots growth. Indeed, lower SPR and increased moisture in treatments with LTM compared to the control can be explained by changes in pore size distribution promoted by SOM, while the higher relative number of small pores due to lower bulk density increases water-holding capacity (Robin et al., 2018). In turn, higher levels of SOM increase aggregation, the stability of soil aggregates, and total pore space, which generally lead to reduced SPR (Tarkiewicz and Nosalewicz, 2005). It is important to highlight that the increases in soil rootability were higher when short-term mulching was used because of the ability of mulch to reduce soil evaporation (Jimenez et al., 2017).

Improvements in soil rootability were reflected in total N uptake by maize in LTM+sN, which was 72% higher than in the treatment with added sN and without mulch. Meanwhile, the positive effect of short-term mulch on N uptake can also be partially explained by the N released by biomass decomposition and increased N availability during the entire maize cycle, which was also verified by Moura et al. (2009). Indeed, accumulated N post-tasseling (ANPT) in STM+sN represented 56% of total N uptake and was 47% higher than in LTM+sN, where NPT represented just 37% of total N uptake. These effects of short- and long-term mulching on N uptake led to an even higher increase in LTM+STM+sN, where total accumulated N was 163% higher than in the compared to the treatment with added sN and no mulching. The differences in N use between treatments can be clearly seen in the CNU results. First, the meager contribution of urea used without mulch (8%) showed that using sN is inviable on uncovered, cohesive tropical soil. Second, as was also reported by Sena et al. (2020), the great contribution of legume mulch (almost 50%) to N uptake showed that its short-term effect is essential to increase N uptake. Finally, combining short- and long-term mulch usage with sN inputs leads to benefits from both inputs, boosting CNU up to 62%.

Both long- and short-term mulching had a positive effect on MGY. However, the size of the effect of the mulches on MGY was heavily reliant on sN. The effect of sN was higher when it was used with LTM (36%) than with STM (15%). In maize, the yield is determined by the harvested kernel number (KN) per unit land area and the average kernel weight (KW) (Wei et al., 2019). Once the number of kernels is established, the final KW becomes a main factor determining maize yield (Yue et al., 2018). KW varies with the rate of kernel growth and the duration of grain-filling. Therefore, a high N supply post-tasseling is beneficial for optimizing grain-filling parameters and improving the KW of maize kernels (Sadras and Egli, 2008). The effect of mulches on KW varied due to differences in their capacity to provide N during the maize growing cycle. Indeed, the fact that KW was higher in STM than in LTM treatments was due to the higher N remobilization and N accumulated at ANPT stage, which resulted in higher MGY.

## 5 Conclusion

Our results showed that mulch of leguminous biomass might avoid land degradation and improve soil fertility in Amazonian periphery. However, there were important differences between effects of short- and long-term mulching. In areas with long-term mulching the interactions between compounds derived of biomass, sN and Ca resulted in higher content of total SOC and base percentage, while short-term mulching-maintained soil moisture, decreased penetration resistance and enhanced N uptake providing biological nitrogen enough to able to replace sN for maize nutrition. Finally, it is worth highlighting that the effects of short- and long-term mulching on maize performance were cumulative. We observed that the two mulching practices alongside synthetic N (LTM+STM+sN treatment) increased accumulated N by 163% and maize grain yield by 125% (4.77 vs 10.78 Mg ha^-1^) compared to cultivation with sN without mulch. Therefore, to prevent degradation of agricultural land, which in turn reduces the risk of new deforestation in Amazonian periphery, the continuously use mulch of leguminous biomass with Ca and sN must be recommended.

## Declaration of Competing Interest

The authors declare that they have no known competing financial interests or personal relationships that could have appeared to influence the work reported in this paper.

## Data Availability

Data will be made available on request.
